# Combinative Treatment of the PARP Inhibitor Olaparib and Antimetastasis Ruthenium(II)–Arene Compound RAPTA-T for Triple-Negative *BRCA1* Wild-Type Breast Cancer Cells

**DOI:** 10.3390/ijms262110613

**Published:** 2025-10-31

**Authors:** Adisorn Ratanaphan

**Affiliations:** Department of Pharmaceutical Chemistry, Faculty of Pharmaceutical Sciences, Prince of Songkla University, Hat-Yai, Songkhla 90112, Thailand; adisorn.r@psu.ac.th; Tel.: +66-0-7428-8867; Fax: +66-0-7442-8239

**Keywords:** combinative treatment, triple-negative breast cancer, BRCA1, olaparib, antimetastasis RAPTA-T, synthetic lethality

## Abstract

To date, breast cancer remains one of the leading causes of death among women worldwide. Although various treatments are used in clinical settings, the efficacy and safety of such treatments are limited by tumor biology factors and patient preferences. Previous studies have shown that triple-negative *BRCA1*-deficient breast cancer is susceptible to DNA-damaging agents, including platinum-based drugs and poly(ADP-ribose) polymerase (PARP) inhibitors, alone or in combination. To address whether the combinative treatment of these DNA-damaging agents can be extended to the triple-negative *BRCA1*-proficient breast cancer population, we investigated the anticancer activity of the well-known FDA-approved PARP inhibitor olaparib in combination with the antimetastatic ruthenium(II)–arene PTA compound RAPTA-T for triple-negative *BRCA1*-competent breast cancer cells (MDA-MB-468 and MDA-MB-231), with consideration of sporadic breast cancer MCF-7 cells. RAPTA-T, olaparib, and the combined agents exhibited a dose-dependent inhibition of breast cancer cell growth in selected breast cancer cells. The combination compound inhibited colony formation most effectively in MDA-MB-468 cells. Additionally, the scratch-wound assay showed that MDA-MB-468 cells migrated more slowly than MCF-7 and MDA-MB-231 cells. The results indicated that the olaparib and RAPTA-T combination can reduce or inhibit the survival, invasion, and metastasis of breast cancer cells. Moreover, the combined agents promoted apoptotic cell death, with a higher percentage of apoptosis observed in MDA-MB-468 cells than in MDA-MB-231 and MCF-7 cells. Olaparib and RAPTA-T also interfered with cell cycle progression, with the greatest inhibition observed in the S and G2/M phases of MCF-7 cells (1.6- and 3.4-fold), followed by MDA-MB-468 cells (1.6- and 1.8-fold) and MDA-MB-231 cells (1.5- and 1.4-fold). Interestingly, MDA-MB-468 cells presented the highest degree of inhibition for *BRCA1* replication and BRCA1 expression. The p53, PARP, and Chk1 proteins were more strongly upregulated in MDA-MB-231 cells than in Ru-untreated control cells. Moreover, the expression levels of protein biomarkers associated with the epithelial-to-mesenchymal transition (EMT), including E-cadherin and SLUG, were remarkably reduced in all tested breast cancer cells. Together, our results show the feasibility of extending the application of PARP inhibitors beyond breast cancer with *BRCA1* mutations and optimizing the combinative treatment of PARP inhibitors with antimetastasis ruthenium-based chemotherapy as new therapeutic approaches for TNBC harboring wild-type *BRCA1*.

## 1. Introduction

**Figure 1:**
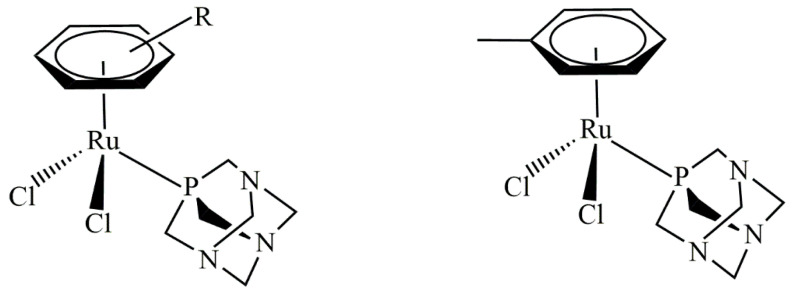
Typical RAPTA structure (**left**) and the structure of RAPTA-T (**right**).

Breast cancer remains one of the leading causes of death among women worldwide. This type of cancer can be classified into five major molecularly distinct subtypes: luminal A, luminal B, basal-like, normal-like, and human epidermal growth factor receptor (HER2) overexpression [1,2,3]. To date, there are a variety of breast cancer treatments used in clinical settings. However, the efficacy and safety of such treatments are limited by tumor biology, particularly for the ‘triple-negative’ breast cancer (TNBC) subtype, which lacks the expression of estrogen receptors (ERs), progesterone receptors (PRs), and HER2 [4,5,6,7]. TNBC accounts for 15–20% of all breast cancers and is frequently diagnosed in African American women. This subtype features pronounced clinicopathological characteristics, such as a high grade, a high rate of cell proliferation, a highly aggressive nature, and less favorable clinical outcomes [8,9,10,11,12]. Presently, there is no standard mode treatment for TNBC patients. Current treatments mostly rely on chemotherapy, patient preferences, and the risks and benefits associated with each treatment protocol [13,14,15,16]. Emerging treatment approaches using DNA-damaging drugs, including platinum-based drugs cisplatin and carboplatin, to treat TNBC are currently being tested in clinical settings based on the *BRCA*ness concept, the postulation that dysfunction of BRCA1 and its associated DNA damage repair pathway sensitizes cells to these drugs or inhibitors [17]. Platinum drugs are commonly used for patients with *BRCA1*-defective TNBC. A decrease in BRCA1 expression in TNBC sensitizes patients to cisplatin [18]. Therefore, platinum-based chemotherapy has improved clinical outcomes for TNBC patients [19]. The molecular pathway of cisplatin-induced apoptotic cell death for TNBC was revealed using triple-negative *BRCA1*-deficient breast cancer HCC1937 cells and triple-negative *BRCA1* wild-type breast cancer MDA-MB-468 cells [20]. Here, the expression of *p63*/*p73* genes seems to reflect a functional consequence related to *BRCA1*-associated tumors [20]. Additionally, increased expression of the p73 protein was apparently observed in MDA-MB-231, MDA-MB-468, and HCC1937 cells after the combination treatment of cisplatin and rapamycin [21,22,23,24]. Moreover, the DNA-damaging platinum drug exhibited a better response in TNBC than non-TNBC patients [25,26,27]. Although widely used in clinical settings, platinum-based drugs have several limitations, including nephrotoxicity, myelosuppression, the induction of secondary mutations, and inevitably acquired resistance [28]. Therefore, several non-platinum-based metal complexes have been developed and assessed as new lead compounds to overcome the drawbacks of classical platinum drugs. In recent years, an increasing number of non-platinum-based compounds have gained significant attention as promising alternatives to traditional anticancer platinum drugs. Specifically, ruthenium(II)–arene compounds containing PTA (PTA = 1,3,5- triaza-7-phosphaadamantane), known as RAPTA, are interesting for their anticancer properties (Figure 1) [29].

RAPTA-T was previously shown to exert antimetastatic activities in an in vitro experimental model. Such effects were clearly observed for invasive MDA-MB-231 breast cancer cells when compared to non-invasive MCF-7 breast cancer cells [30,31,32,33,34]. It was also reported that RAPTA-T perturbs the BRCA1 RING protein domain secondary structure, resulting in the displacement of zinc ions, eventually preventing BRCA1-mediated E3 ubiquitin ligase activity [35]. RAPTA-T inhibits the metastasis pathway, including detachment from the primary cancerous cell, migration, and re-attachment steps that are mediated through interactions with the extracellular matrix [36,37]. A recent study further showed that RAPTA-T causes much more cellular *BRCA1* damage in triple-negative *BRCA1*-defective HCC1937 breast cancer cells than in sporadic *BRCA1* wide-type MCF-7 breast cancer cells, leading to a reduction in both *BRCA1* mRNA expression and the BRCA1 protein in both cell lines [38].

Poly(ADP-ribose) polymerase (PARP) is a DNA repair protein that plays an important role in repairing single-strand DNA breaks (SSBs), preventing the generation of the most cytotoxic double-strand DNA breaks (DSBs) [39]. Cells that contain *BRCA1/2* gene mutations possess an inherent deficiency in the homologous recombination (HR) repair of DSBs. This deficiency results in synthetic lethality between *BRCA1/2* deficiency and PARP inhibition [40,41]. Accordingly, PARP inhibitors (PARPi) were developed as single-agent treatments for *BRCA1/2*-deficient breast, ovarian, and prostate cancers [42]. Deletion of the *PARP* gene in experimental cell models alongside high levels of SSBs results in the accumulation of DSBs, cell-cycle arrest, and/or cell death [43,44,45,46]. Several PARP inhibitors (PARPi) have been preclinically and clinically evaluated. In 2018, PARPi olaparib (Lynparza^®^) was approved for the clinical treatment of *BRCA*-mutated HER2-negative metastatic breast cancer and germline *BRCA*-mutated metastatic pancreatic cancer, as well as the maintenance of *BRCA*-mutated advanced epithelial ovarian cancers. The rational combination of a DNA-damaging agent with a PARP inhibitor was hypothesized to induce synergistic activity [47,48,49,50,51]. Today, this concept increasing represents a novel therapeutic approach that could be applied to other breast cancer subtypes, irrespective of *BRCA* status. Synergism can be observed in the combination treatment of olaparib with some alkylating agents in various cancers, independent of their *BRCA* status [51]. However, the combination treatment of olaparib and ruthenium-based compounds was only recently studied as a new therapeutic approach [52]. A recent study on the combination of ruthenium(II) metallointercalators with olaparib showed synergy in *BRCA*-proficient TNBC. In addition, combinative treatment using a glutathione transferase (GSTP-1) inhibitor-derived RAPTA compound (RAPTA-EA1) and olaparib significantly suppressed the expression levels of BRCA1 protein in triple-negative *BRCA1* wild-type breast cancer cells [53]. These results demonstrate that drug susceptibility in triple-negative *BRCA1* competent breast cancer cells is closely associated with the reduced ability of BRCA1 protein to repair cellular DNA damage after drug exposure. Simultaneously, olaparib itself interferes with PARP function, leading to cancer cell death via synthetic lethality [54]. However, to address whether the combinative treatment of such DNA-damaging agents can be extended to the triple-negative *BRCA1*-proficient breast cancer population, the anticancer activity of the well-known PARP inhibitor olaparib in combination with the antimetastatic ruthenium(II)–arene PTA compound RAPTA-T was investigated to treat triple-negative *BRCA1*-competent breast cancer cells (MDA-MB-468 and MDA-MB-231), with consideration of sporadic breast cancer MCF-7 cells. This study could provide insights into alternative treatments for TNBC harboring wild-type *BRCA1*.

## 2. Results

### 2.1. Cytotoxic Effects of RAPTA-T or Olaparib as a Single Agent and Combination Compound on Human Breast Cancer Cells

**Figure 2:**
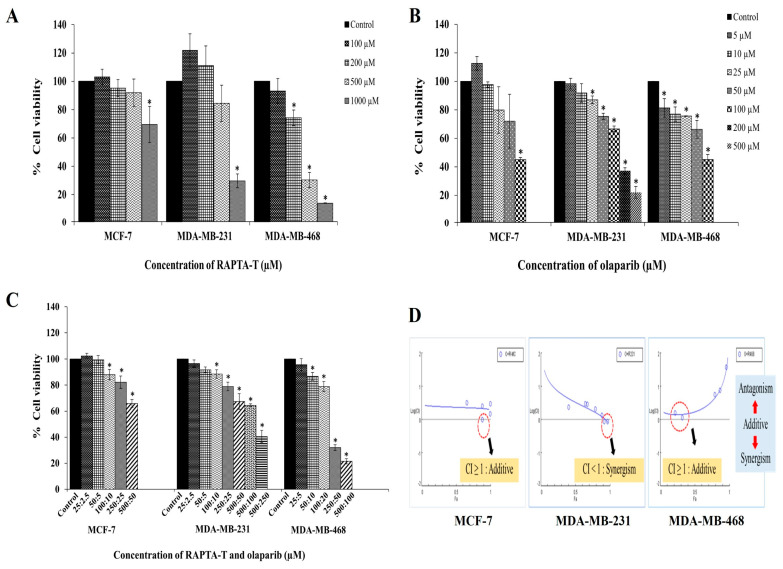
Cytotoxic effects of RAPTA-T (**A**), olaparib (**B**), and their combination (**C**) on MCF-7, MDA-MB-231, and MDA-MB-468 cells. Cells were treated with various concentrations of RAPTA-T and/or olaparib for 48 h, and cell viability was determined by the MTT assay. (**D**) The combinatorial of RAPTA-T and olaparib on BRCA-related cancer cells were further evaluated using the combination index (CI) method according to [55]. Data are presented as mean ± SD from three independent experiments. Statistically significant differences compared with the control are indicated by * *p* < 0.05.

Using MTT assays, the cytotoxicity effects of RAPTA-T and olaparib as single agents in three breast cancer cell lines, MCF-7, MDA-MB-231, and MDA-MB-468 cells were investigated. The results showed a dose-dependent inhibition of cell growth, increasing in concentration with a decrease in cell viability in all tested cell lines (Figure 2A,B).

MDA-MB-468 cells were significantly more sensitive to RAPTA-T than MCF-7 and MDA-MB-231 cells. The IC_50_ values for all treated cells are reported in Table 1. Treatment with RAPTA-T and olaparib appeared more active against MDA-MB-468, MDA-MB-231, and MCF-7 cells. In the present study, RAPTA-T or olaparib alone exhibited a dose-dependent decrease in cell viability, while combination treatment using both RAPTA-T and olaparib yielded reduced viability in these cell lines (Figure 2C).

### 2.2. Combination Index (CI)

The combination index (CI) was used to assess the effects of RAPTA-T and olaparib (at its IC_50_) in a combination treatment using the Chou–Talalay method [55]. The results showed a combination index (CI) of CI ≥ 1 (additive) in MCF-7 cells, CI < 1 (synergism) in MDA-MB-231 cells, and CI ≥ 1 (additive) in MDA-MB-468 cells, as shown in Figure 2D.

### 2.3. Clonogenic Assay

**Figure 3:**
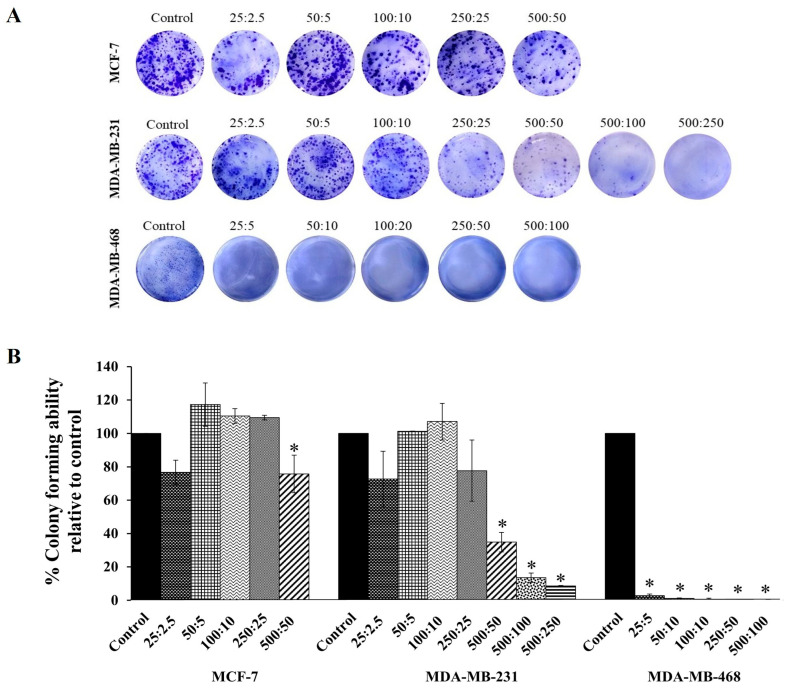
The colony forming efficiency assay of RAPTA-T and olaparib combination compound on MCF-7, MDA-MB-231, and MDA-MB-468 cells. (**A**) Representative images of colonies formed after cells were seeded in 6-well plates, treated with a combination of RAPTA-T and olaparib for 48 h, reseeded in compound-free medium, and cultured for 10 days, visualized by staining with 1% Coomassie brilliant blue for 1 h. (**B**) Quantitative analysis of colony forming ability relative to the control. Data are presented as mean ± SD from three independent experiments. Statistically significant differences compared with the control are indicated by * *p* < 0.05.

Based on the cytotoxicity effects shown in Figure 2, the combination compound containing RAPTA-T and olaparib was subjected to a clonogenic assay to determine whether growth-inhibitory concentrations of the combination compound could inhibit colony formation. The results showed that at the same concentration, this compound more significantly inhibited the colony formation of MDA-MB-468 cells, and, to a lesser extent, MDA-MB-231 and MFC-7 cells, compared with the control (Figure 3), which agrees with the MTT results. Additionally, when compared with the control and all treated cells, the colony formations in MDA-MB-468 cells treated with the combination compound were generally smaller in size than those in MDA-MB-231 and MCF-7 cells.

### 2.4. Scratch-Wound Assay

**Figure 4:**
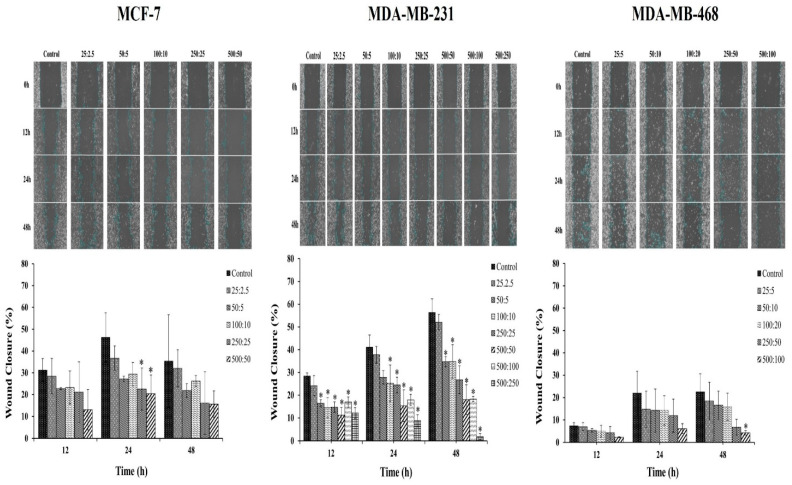
Effect of the combination compound of RAPTA-T and olaparib on the migration of MCF-7, MDA-MB-231, and MDA-MB-468 cells, as determined by a scratch-wound assay. About 5 × 105 cells were seeded into a 6-well plate and left in the incubator until a confluent monolayer of cells formed. Cells were scratched with a sterile 200 μL pipet tip, a straight scratch was made in each well and washed with PBS twice. Cells were then treated with a combination of RAPTA-T and olaparib. The migration of the cells in the wound scratch area was analyzed and photographed at 0, 12, 24, and 48 h. The images captured were then analyzed using the ImageJ software version 1.54g. The percentage of wound scratch closure was determined by measuring the reduction in the area of the wound at each time point compared to the area at 0 h (100%). Data are presented as mean ± SD (* *p* ≤ 0.05).

The effect of the combination compound containing RAPTA-T and olaparib on the migration of breast cancer cells was further studied. Breast cancer cells possess the ability to metastasize from the original tumor site to other organs, leading to the spread of cancer. Therefore, a scratch-wound assay was performed to determine the effects of the RAPTA-T and olaparib combination compound on the migration of MCF-7, MDA-MB-231, and MDA-MB-468 cells. The results indicated that the combination compound offers significant inhibitory effects on the migration of all breast cancer cells (Figure 4). Particularly, the migration of MDA-MB-468 cells offered greater inhibitory effects than that of MCF-7 and MDA-MB-231 cells after treatment with the combination compound for 48 h. These results and those of the clonogenic assay demonstrate the potential of the combination compound using RAPTA-T and olaparib to reduce or inhibit the survival, invasion, and metastasis of breast cancer cells.

### 2.5. Apoptosis Annexin V/FITC Assay

**Figure 5:**
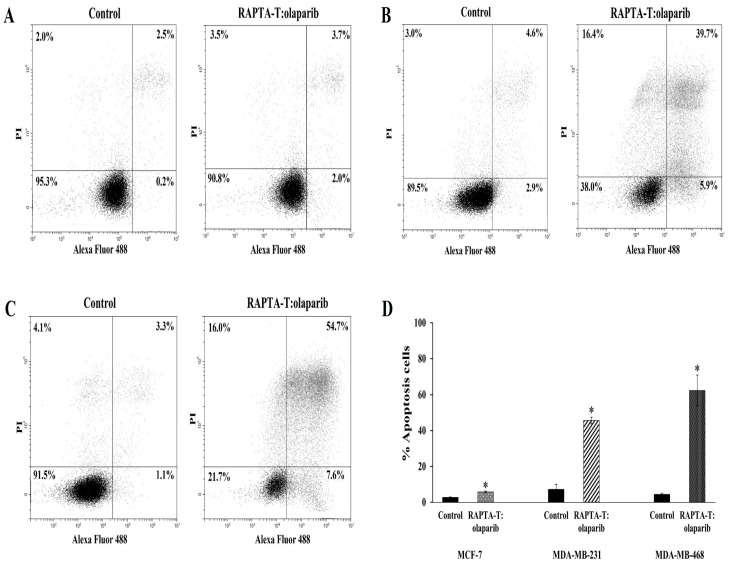
Effect of the combination compound of RAPTA-T and olaparib on apoptosis cell death of MCF-7 (**A**), MDA-MB-231 (**B**) and MDA-MB-468 (**C**) cells. Cells were incubated in combination compound (IC_50_) for 48 h. The percentage of apoptotic cells was detected by analyzing for the Annexin V-FITC and PI binding using flow cytometry presented by the sum of the early apoptotic presented and late apoptotic cells (Annexin V+/PI+). The standard error of experiments realized in triplicate was plotted as shown in a bar graph (**D**). Results are mean ± standard deviation of three independent experiments (* *p* < 0.05).

The effects of the RAPTA-T and olaparib combination compound at IC_50_ on the apoptotic cell death were analyzed using flow cytometry. The induction of apoptotic cells was determined by the sum of both early and late apoptotic cells (Figure 5). A greater increase in the percentage of apoptotic cells was observed in MDA-MB-468 (62.3%) than in MDA-MB-231 (45.6%) and MCF-7 (5.7%).

### 2.6. Cell Cycle Analysis

**Figure 6:**
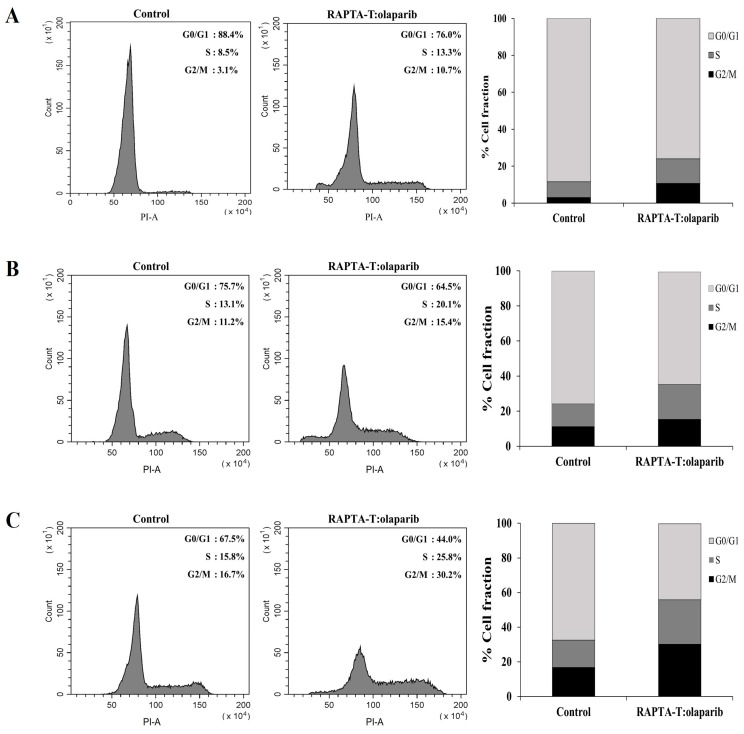
Effect of the combination compound containing RAPTA-T and olaparib on the cell cycle distribution of MCF-7 (**A**), MDA-MB-231 (**B**) and MDA-MB-468 (**C**) cells. Cells were exposed to the combination compound at IC_50_ for 48 h at 37 °C. The DNA content was measured by PI staining. The percentage of cells in the G0/G1, S, and G2/M phases were analyzed using flow cytometry. The experiment was conducted in triplicate.

The effects of the RAPTA-T and olaparib combination on cell cycle distribution and apoptotic cells were estimated via propidium iodide (PI) staining using flow cytometric analysis. As observed from the results, the population of cells in S and G2/M cell cycle phase increased in three breast cancer cell lines compared with the control. The combination compound at IC_50_ showed an effect to arrest at the S and G2/M phase of MCF-7 (1.6 and 3.4-fold) more than MDA-MB-468 (1.6 and 1.8-fold) and MDA-MB-231 (1.5 and 1.4-fold), respectively, with a greater percentage of apoptotic cells was observed in the MCF-7 cells than in the MDA-MB-468 and MDA-MB-231 cells (Figure 6).

### 2.7. Immunofluorescence Assay

**Figure 7:**
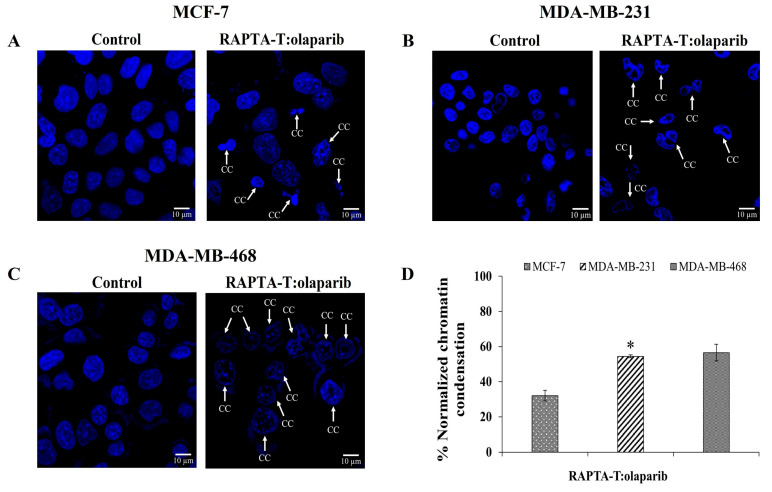
DAPI-stained nuclei of MCF-7 (**A**), MDA-MB-231 (**B**) and MDA-MB-468 (**C**) cells were shown. Cells were treated with the combination compound using RAPTA-T and olaparib (IC_50_) for 48 h at 37 °C, the arrow represents chromatin condensation in the cells after the treatment. The percentage of normalized chromatin condensation was calculated by (number of cells with condensed chromatin/(number of cells with condensed chromatin + number of cells with intact chromatin) × 100) (**D**). Results are mean ± standard deviation of three independent experiments (* *p* < 0.05).

To examine whether combination RAPTA-T and olaparib-induced cell death was related to apoptosis, cells incubated with IC_50_ for 48 h were stained with 4, 6-diamidio-2-phenylindole (DAPI) to investigate chromatin condensation. The RAPTA-T and olaparib combination compound induced nuclei morphological changes, shrinkage, and fragmentation, which are characteristics of nuclear condensation and indicative of apoptosis in all cancer cells. The percentage of the normalized chromatin condensation is shown in Figure 7. MDA-MB-468 cells showed more condensed chromatin (56.54%) compared with MDA-MB-231 (54.43%) and MCF-7 (32.05%) cells.

### 2.8. Quantitative PCR (QPCR) for Cellular BRCA1 Damage

**Figure 8:**
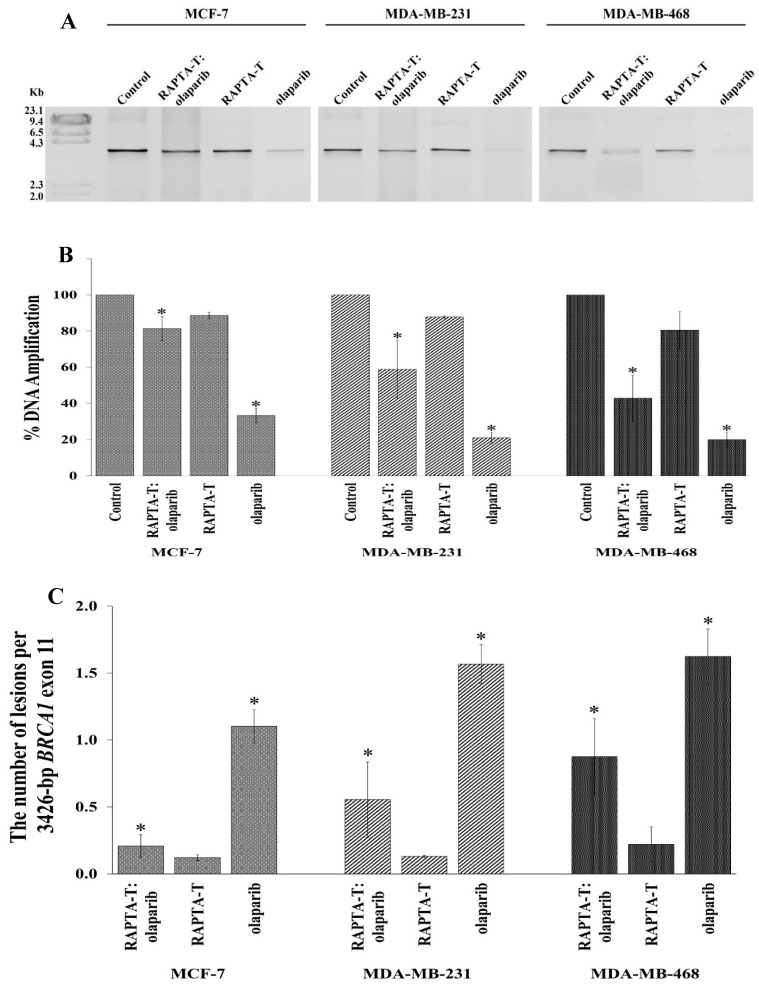
Cellular *BRCA1* damage in MCF-7, MDA-MB-231 and MDA-MB-468 cells. (**A**): Cells were incubated with the combination compound of RAPTA-T and olaparib for 48 h. Genomic DNA of the single agent-treated, combination-treated and control (untreated) cells was isolated and the 3426 bp *BRCA1* exon 11 fragment was then amplified by the PCR reaction, and the PCR products were electrophoresed on 1% agarose gel. The gel was stained with ethidium bromide and visualized under UV illumination. M stands for λ-HindIII digested marker. (**B**): Amplification (%) products were quantified band intensity using an Image Quant™ LAS 500. (**C**): Lesion frequency per the 3426 bp fragment of the *BRCA1* exon 11 induced by the combination compound of RAPTA-T and olaparib calculated by the Poisson equation. Results are mean ± standard deviation of three independent experiments (* *p* < 0.05).

The effects of the single agent and combination treatment using RAPTA-T and olaparib on cellular BRCA1 damage in MCF-7, MDA-MB-231, and MDA-MB-468 cells was investigated using a quantitative PCR-based assay (QPCR). After exposure to the single and combination treatments for 48 h, genomic DNA was extracted. The results showed that BRCA1 amplification (%) was reduced in all tested cells compared with the control (Figure 8A,B). Olaparib yielded the greatest inhibition of BRCA1 amplification, followed by the combination treatment and RAPTA-T. Notably, the combination treatment also offered a higher degree of *BRCA1* inhibition than RAPTA-T itself but a lower degree than the results when using olaparib alone. In addition, the replication blocking capacity of the *BRCA1* gene induced by the combination compound was significantly higher in MDA-MB-468 (57.16%) cells than in MDA-MB-231 (41.13%) and MCF-7 (18.66%) cells.

Lesion induction within the 3426 bp fragment of the BRCA1 exon 11 can be calculated using the Poisson equation [38]. The lesion frequency according to the 3426 bp fragment of the *BRCA1* exon 11 in MCF-7, MDA-MB-231, and MDA-MB-468 cells treated with the RAPTA-T and olaparib compound is shown in Figure 8C. The combination compound exhibited approximately twofold more lesions in MDA-MB-468 cells than in MDA-MB-231 and MCF-7 cells under equivalent experimental conditions.

### 2.9. Protein Extraction and Western Blotting Analysis

**Figure 9:**
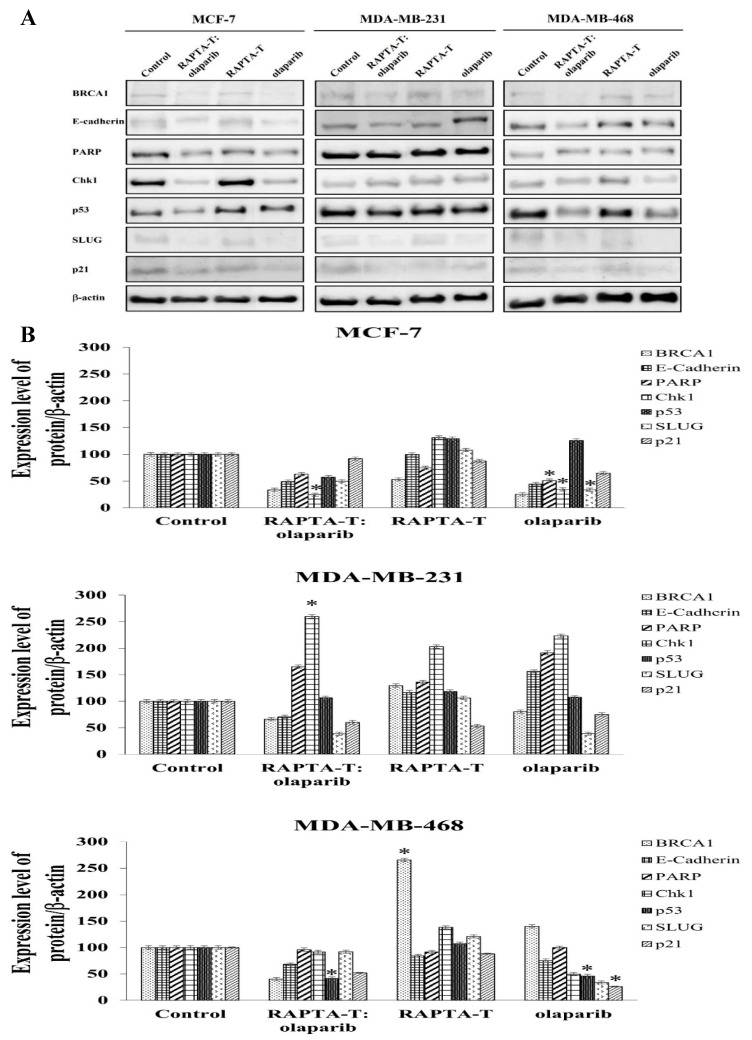
Effect of the single agent and combination compound using RAPTA-T and olaparib on protein expression. (**A**) Whole-cell proteins were prepared from the cells treated with IC_50_ of the compound for 24 h. Cellular protein extracts were separated on a 6%, 8% and 12% SDS-PAGE gel, transferred onto nitrocellulose membrane, and immunoblotted with the primary antibodies. The protein loading of each sample was controlled by β-actin expression. (**B**) The percentage of relative expression of protein was shown for each cell line. Experiments were performed in duplicate. Statistically significant differences from the untreated control are indicated by * *p* < 0.05.

To examine the effects of the RAPTA-T and olaparib combination and single-agent treatments on protein expression, Western blot analysis was performed after 24 h of treatment. Whole-cell protein extracts were detected using specific antibodies, the results of which are shown in Figure 9. The combination treatment of RAPTA-T and olaparib suppressed BRCA1 expression better than the single agent treatment in all three cell lines. Additionally, RAPTA-T slightly reduced the BRCA1 protein levels in these tested cells.

Examining p53 protein expression, the combination treatment of RAPTA-T and olaparib was p53-upregulated in MDA-MB-231 cells but downregulated in MCF-7 and MDA-MB-468 cells. For single-agent treatments, both RAPTA-T and olaparib were p53-upregulated in all three cell lines, except for olaparib, which was downregulated in MDA-MB-468 cells.

Both the combination and single-agent treatments showed an ability to suppress expression of the p21 protein in all three cell lines. The combination treatment was observed to yield the greatest decrease in relative expression in MDA-MB-468 cells, followed by MDA-MB-231 and MCF-7 cells.

Further investigations into the effects of treatment using the RAPTA-T and olaparib compound, as well as the single-agent treatments, were conducted alongside an assessment of DNA repair. In the evaluation of PARP expression, both the combination and single-agent treatments demonstrated that MDA-MB-231 cells had a greater ability to increase expression of the PARP protein. In contrast, the PARP protein levels in MCF-7 and MDA-MB-468 cells decreased under both treatments.

The regulation of the DNA damage checkpoint was further studied by examining expression of the Chk1 protein. The results showed that the expression of Chk1 protein increased in MDA-MB-231 cells under both the combination and single-agent treatments. In contrast, the combination treatment in the other cell lines showed a reduction in Chk1 protein expression, with a greater reduction observed in MCF-7 cells than in MDA-MB-468 cells. For single-agent treatments in MCF-7 and MDA-MB-468 cells, RAPTA-T enhanced Chk1 expression, while olaparib caused a reduction in more than 50% in protein expression.

Whether the combination and single agents affected the expression of proteins involved in the epithelial-to-mesenchymal transition (EMT), including E-cadherin and SLUG was further investigated [56]. The results showed that the levels of E-cadherin protein under the combination treatment using RAPTA-T and olaparib decreased in all three cell lines. The expression levels were found to be the highest in MDA-MB-231 cells (71%), with lower levels in MDA-MB-468 (68%) and MCF-7 cells (49%). The use of RAPTA-T or olaparib as single agents also reduced the protein expression in MCF-7 and MDA-MB-468 cells. However, in MDA-MB-231 cells, both single agents increased the expression levels of E-cadherin. The protein expression of SLUG was reduced in these cell lines after exposure to the combination and single-agent treatments. Under the combination treatment, MDA-MB-231 cells exhibited the greatest reduction in protein levels, followed by MCF-7 cells and MDA-MB-468 cells. In contrast, the single-agent RAPTA-T treatment showed an increase in SLUG protein expression, with the highest relative increase observed in MDA-MB-468 cells, followed by that in MDA-MB-231 and MCF-7 cells.

## 3. Discussion

In this study, the ability of RAPTA-T and olaparib in combination and as single agents to inhibit triple-negative BRCA1 wild-type MDA-MB-231 and MDA-MB-468 cells compared with sporadic *BRCA1* wild-type MCF-7 breast cancer cells was investigated. Under both the single-agent and combination treatments, RAPTA-T and olaparib yielded a dose-dependent inhibition of cell viability in all three cell lines, based on an MTT assay. In previous research, RAPTA-T showed antimetastatic [30] and antiangiogenic [31] properties. RAPTA-T exhibited greater effects on highly invasive metastatic MDA-MB-231 cells, compared to non-invasive MCF-7 cells [57,58]. It was also found that the RAPTA-T compound works on molecular targets other than DNA, implying a biochemical mode of action profoundly different from that of classical platinum-based anticancer drugs [59,60,61,62]. Additionally, it was discovered that ruthenium might interact with sporadic BRCA1 wild-type MCF-7 and BRCA1-defective HCC1937 cells, potentially due to the accumulation of ruthenium in the cytoplasm via the transferrin membrane receptor. This interaction may interfere with specific cellular proteins involved in signal transduction pathways, cell adhesion, and migration processes, which are likely critical to ruthenium’s mode of action [34,63,64]. The synergy between RAPTA-T and olaparib was found that the combination index (CI) for MCF-7 and MDA-MB-468 was additive (CI ≥ 1), while that for MDA-MB-231 was synergistic (CI < 1).

To examine the effectiveness of the combination treatment in breast cancer cell lines, we conducted clonogenic assays showing that the combination of RAPTA-T and olaparib at various doses independently retained the ability to inhibit single cells from forming into colonies. Notably, MDA-MB-468 cells inhibited colony formation at low concentrations and generated smaller colony sizes. To further examine the effects of combination treatment on the ability of cancer cells to metastasize and grow, a scratch-wound assay was conducted. The migration of invasive triple-negative breast cancer cells and MDA-MB 468 cells was slower than that of MCF-7 and MDA-MB-231 cells at the same time points. The results of the cell growth, clonogenic, and scratch-wound assays demonstrated the potential of the combination treatment to reduce or inhibit the survival, invasion, and metastasis of breast cancer cells. These results may be associated with levels of the transcription factor SLUG protein, which regulates the epithelial-to-mesenchymal transition (EMT) [56].

The present results demonstrate that the combination treatment promoted the apoptotic process in breast cancer cells, with a greater percentage of apoptotic cells observed in MDA-MB-468 cells compared to that in MDA-MB-231 and MCF-7 cells. Cell cycle progression showed arrest in the S and G2/M phases of MCF-7 (1.6- and 3.4-fold), MDA-MB-468 (1.6- and 1.8-fold), and MDA-MB-231 (1.5- and 1.4-fold) cells. Moreover, the combination of RAPTA-T and olaparib induced nuclear morphological changes and fragmentation, which are characteristic of nuclear condensation and indicative of apoptosis in all cancer cells. MDA-MB-468 cells exhibited a higher percentage of condensed chromatin (57%) compared to that in MDA-MB-231 (54%) and MCF-7 (32%) cells.

The level of BRCA1 amplification was inversely proportional to the quantity of DNA lesions within the specified DNA region. Olaparib alone yielded a greater inhibition of DNA synthesis than both the combination treatment and single agent RAPTA-T in all breast cell lines. Single agent RAPTA-T exhibited lower inhibition of DNA synthesis than that under the combination treatment. The combination treatment using RAPTA-T and olaparib corresponded to approximately twofold more DNA lesions in MDA-MB-468 cells compared to those observed in MDA-MB-231 and in MCF-7 cells. In addition, the ruthenium compound exhibited an approximately threefold greater ability to suppress expression of the BRCA1 protein and a twofold greater ability to suppress expression of the p53 protein compared with the Ru-untreated control in MCF7 cells. Further analyses of the compound containing RAPTA-T and olaparib, as well as the single agents, revealed that the BRCA1 protein expression levels decreased in all cells subjected to the combination treatment, with MCF-7 cells presenting approximately 70% expression inhibition. This inhibition was more effective than that of the single agents, RAPTA-T and olaparib, compared with the control. The combination treatment inhibited p53 protein biosynthesis in MCF-7 and MDA-MB-468 cells but increased in MDA-MB-231 cells. The biosynthesis of p21 protein was inhibited in all three cell lines. In terms of the DNA repair-related protein PARP, MDA-MB-231 cells exhibited increased protein biosynthesis under both the combination and single agent treatments, whereas MCF-7 and MDA-MB-468 cells showed reduced PARP expression. The expression levels of the Chk1 protein, which is involved in DNA damage responses was also studied. The results showed an increase in Chk1 protein levels in MDA-MB-231 cells, while MCF-7 and MDA-MB-468 cells presented a reduction in protein levels. In addition, the expression levels of E-cadherin and SLUG proteins, which are involved in characteristic cell changes through the epithelial-to-mesenchymal transition (EMT) process, was analyzed for migration and metastasis inhibition in the three types of breast cancer cells. The results showed that the expression levels of both E-cadherin and SLUG proteins decreased in all cells treated with both the compound and single agents. In addition, many microRNAs have been implicated in breast cancer migration, invasion and metastasis [65]. Together, the present data show that expanding the application of PARP inhibitors beyond breast cancer with *BRCA1* mutations (irrespective of *BRCA1* status) and optimizing combinative treatment using PARP inhibitors with antimetastasis ruthenium-based chemotherapy could represent a new therapeutic approach for TNBC harboring wild-type *BRCA1* [66].

## 4. Materials and Methods

### 4.1. Chemicals and Reagents

The organometallic ruthenium(II)–arene compound [Ru (*η*^6^-toluene) Cl_2_ (PTA)], RAPTA-T was provided by Prof. Paul J. Dyson, Institute of Chemical Sciences and Engineering, Swiss Federal Institute of Technology (EPFL), CH-1015 Lausanne, Switzerland. Olaparib was purchased from Sigma-Aldrich, St. Louis, MO, USA. Stock solutions of RAPTA-T (10 mM) were prepared in deionized water, while olaparib (50 mM) was prepared in 100% dimethyl sulfoxide (DMSO) and further diluted using DMEM or an RPMI 1640 medium. A final DMSO concentration of 0.5% was employed in the cell analyses.

### 4.2. Cell Lines

The MCF-7 (sporadic BRCA1 wild-type), MDA-MB-231 (TNBC BRCA1 wild-type), and MDA-MB-468 (TNBC BRCA1 wild-type) breast cancer cell lines were obtained from the American Type Culture Collection (ATCC), Rockville, MD, USA. MCF-7 and MDA-MB-231 cells were cultured in Dulbecco’s modified Eagle’s medium (DMEM) (Sigma-Aldrich, St. Louis, MO, USA), while MDA-MB-468 cells were cultured in an RPMI 1640 medium (Sigma-Aldrich, St. Louis, MO, USA). Media were supplemented with 10% fetal bovine serum (FBS) and 1% penicillin-streptomycin, without phenol red. All cell lines were cultured at a constant temperature of 37 °C in a humidified 5% carbon dioxide (CO_2_) atmosphere.

### 4.3. MTT Proliferation Assay

About 1 × 10^4^ cells were seeded into each well of a 96-well culture plate and incubated for 48 h at 37 °C in a 5% CO_2_ incubator. The culture medium was then removed, and 200 µL of fresh culture medium with various concentrations of RAPTA-T (0–1000 μM), olaparib (0–500 μM), or a combination treatment of RAPTA-T and olaparib was added. The breast cancer cells were further incubated for 48 h. After incubation, each well was washed twice with 100 μL of phosphate-buffered saline (PBS). A solution of 3-(4,5-dimethylthiazol-2-yl)-2,5-diphenyl tetrazolium bromide (MTT) (0.5 mg/mL) was added to each well and incubated for an additional 4 h. The MTT solution was then gently removed, and DMSO (200 µL) was added to solubilize the crystals of the purple formazan formed. The absorbance of each well was spectrophotometrically measured at 570 nm using an automated microplate reader, and the cell viability (%) was calculated as follows:

Cell viability (%) = [(absorbance of the treated cells/absorbance of the control) × (the untreated cells)] × 100

The percentage of cell viability was plotted against various concentrations of RAPTA-T or olaparib to determine the 50% inhibitory concentration (IC_50_), defined as the concentration of RAPTA-T or olaparib that inhibited cell viability by at least 50% compared to that under the control condition.

### 4.4. Drug Interaction Analysis

Dose–effect curves for the single agent and combination treatments were generated using the MTT assay. Additive, antagonistic, or synergistic interactions were assessed using the combination index (CI) with the Chou–Talalay method [55], calculated using the CompuSyn program (ComboSyn, Inc., Paramus, NJ, USA).

### 4.5. Clonogenic Survival Assay

About 1 × 10^5^ cells were seeded into a 6-well plate and allowed to adhere for 48 h. The cells were then treated with a combination of RAPTA-T and olaparib for 48 h. After treatment, the solutions were removed. Next, the cells were trypsinized, reseeded in a new 6-well plate at a density of 1 × 10^3^ cells/well, and cultured in compound-free medium. The cells were incubated for 10 days to allow colony formation. The cells were then fixed for 30 min using a fixation solution [(methanol/glacial acetic acid, 3:1 (*v*/*v*)] and stained with 1% Coomassie blue for 1 h. The staining solution was washed with water, and images were taken with a digital camera. The images were analyzed using the ImageJ software.

### 4.6. Scratch-Wound Assay

About 5 × 10^5^ cells were seeded into a 6-well plate and left in the incubator until a confluent monolayer of cells formed. Using a sterile 200 μL pipet tip, a straight scratch was made in each well and washed with PBS twice. Cells were then treated with a combination of RAPTA-T and olaparib. The migration of the cells in the wound scratch area was analyzed and photographed at 0, 12, 24, and 48 h. Images were photographed at 10× magnification using a microscope attached to a digital camera, and the images captured were then analyzed using the ImageJ software. The percentage of wound scratch closure was determined by measuring the reduction in the area of the wound at each time point compared to the area at 0 h (100%).

### 4.7. Apoptosis Annexin V/FITC Assay

About 10^5^ cells were seeded into a 6-well plate and allowed to adhere for 24 h. Cells were treated with a combination treatment of RAPTA-T and olaparib for 48 h at 37 °C under 5% CO_2_. After treatment, the cells were trypsinized, washed twice with cold PBS, and then centrifuged at 300 g for 5 min. The supernatant was discarded and 100 μL of 1× Annexin-binding buffer was added to resuspend the cell pellets. Alexa-Fluor 488 Annexin-V (5 μL) and 100 μg/mL of PI (1 μL) were added to each cell suspension and mixed gently, and further incubated for 15 min at room temperature in the dark. A solution of 1× Annexin V binding buffer (400 μL) was subsequently added and mixed gently. Analysis of Annexin-V binding was performed using CytoFlex flow cytometer (Beckman Coulter, Brea, CA, USA). The percentage of apoptotic cells was calculated from the total 20,000 cells.

### 4.8. Cell Cycle Analysis

The cells were seeded at 1 × 10^6^ cells per well in 6-well plates and grown at 37 °C under 5% CO_2_ until approximately 80% confluence. The medium was removed and the cells were then treated with the combination compound of RAPTA-T and olaparib (IC_50_) at for 48 h at 37 °C under 5% CO_2_. After treatment, the cells were harvested by trypsinization, washed with cold PBS twice, and then centrifuged at 300 g for 5 min at 4 °C. The cells were collected and fixed with cold 70% ethanol at least overnight at −20 °C. Following fixation, the cells were centrifuged at 500 g for 5 min, and the resulting pellets were washed with PBS twice. The samples were then resuspended in 1 mL of PBS containing RNase (100 μg/mL), PI (50 μg/mL), and Triton-X-100 (0.1%). After being incubated at room temperature for 30 min, samples were acquired and analyzed with a CytoFlex flow cytometer (Beckman Coulter, Brea, CA, USA). The DNA content was measured by counting 20,000 cells, and the cell cycle distribution was analyzed using the CytExpert software version 2, which represents the percentages of cells at the G0/G1, S, and G2/M phases.

### 4.9. Immunofluorescence Assay

Cells were seeded at a density of 1 × 10^5^ cells per well in a 6-well plate and allowed to adhere for 48 h at 37 °C under 5% CO_2_. Cells were then treated with the combination compound of RAPTA-T and olaparib (IC_50_) for 48 h, fixed with 4% paraformaldehyde for 30 min, and permeabilized with 0.2% Triton X-100. The cells were incubated with a blocking reagent [3% bovine serum albumin (BSA) in Tris-buffered saline (TBS) with 0.1% Tween x20 (TBST)] for 1 h at room temperature, and then their nuclei were counterstained with 4, 6-diamidio-2-phenylindole (DAPI 1 μg/mL).

### 4.10. Quantitative PCR (QPCR) for Cellular BRCA1 Damage

Cells were seeded at 1 × 10^6^ cells per well in 6-well plates and treated with either the single agent (IC_50_) or a combination compound of RAPTA-T and olaparib (IC_50_) for 48 h at 37 °C under 5% CO_2_. After treatment, solutions were removed, and cells were trypsinized and washed with cold PBS twice. The cells were collected, and the genomic DNA was isolated, and the 3426 bp fragment of the *BRCA1* exon 11 of the cells was then amplified in a PCR reaction. Briefly, PCR reactions were carried out in a total volume of 50 μL containing 400 ng of a treated genomic DNA template, 0.5 µM of forward (5′-GCCAGTTGGTTGATTTCCACC-3′) and reverse primers (5′-GTAAAATGTGCTCCCCAAAAG -3′), 200 µM of each dNTP, 1.5 units of Phusion Hot Start DNA polymerase, 3 mM MgCl_2_, 1× PhusionT™ GC Buffer, and sterile water, comprising a total volume of 50 μL. The 3426 bp fragment of the *BRCA1* exon 11 in the cells was then amplified using thermal cycle conditions as follows: 3 min at 94 °C; 30 cycles of 30 s at 94 °C, 45 s at 60 °C, 2 min at 72 °C, and a final extension for 7 min at 72 °C. The PCR products were separated via 1% agarose gel electrophoresis, stained with ethidium bromide, and visualized under UV light. The quantitative PCR (QPCR) method was then used to assess the DNA polymerase blocking effect of the damaged DNA. The amplification products were quantified using an Image Quant™ LAS 500, and the amount of DNA amplification (%) was plotted in combination. The number of lesions per 3426 bp fragment of the *BRCA1* exon 11 was estimated with the Poisson equation [38]. Experiments were conducted in triplicate:

S = −ln Ad/A

where S is the lesion frequency per strand, A is the absorbance produced from a given amount of the non-damaged DNA template, and Ad is the absorbance produced from a given amount of the damaged DNA template induced by the single agent or combination compound. Therefore, Ad/A gives the fraction of non-damaged templates at a given dose.

### 4.11. Protein Extraction and Western Blotting Analysis

After incubation for 24 h with the single agent and combination compound of RAPTA-T and olaparib (IC_50_) at 37 °C, the cells were washed twice with PBS, harvested, and lysed for 5 min in wells containing 200 μL of a lysis buffer (50 mM Tris (pH 8.0), 5 mM EDTA, 5 mM NaCl, 100 mM PMSF, 2% SDS, 1% Triton X-100). Then, the cells were placed on ice. The lysate proteins were isolated via centrifugation at 16,000× *g* for 10 min under a temperature of 4 °C. The concentration of the crude protein in each sample was determined using a Bradford assay. Protein samples of 20 ug were mixed with an electrophoresis buffer and boiled for 10 min followed by separation using electrophoresis on 6%, 8%, and 12% SDS polyacrylamide gel. Then, using a semi-dry electroblotter, separated proteins in the gel were transferred to a nitrocellulose membrane through a transfer buffer (14.4 g of glycine, 3.03 g of Tris base, 200 mL of methanol, and double distilled water to a final volume of 1 L) under a current of 400 mA for 6 h. The nitrocellulose membrane was incubated with shaking for 2 h in 5% bovine serum albumin (BSA), the blocking reagent buffer in Tris-buffered saline with 0.1% Tween-20 (TBST). After removing the blocking reagent buffer, the nitrocellulose membrane was further incubated overnight at 4 °C with an appropriate dilution (1:1000) in a TBST buffer containing a primary antibody, anti-BRCA1 (A8X9F) rabbit antibody (14823, Cell Signaling, Danvers, MA, USA), anti-E-cadherin mouse antibody (AF748 R&D system, Minneapolis, MN, USA), anti-PARP rabbit antibody (9532 Cell Signaling), anti-Chk1 (E9M4D) rabbit antibody (37010S Cell Signaling), anti-p53 mouse antibody (HAF1355, R&D Systems), anti-SLUG (C19G7) rabbit antibody (9585 Cell Signaling), and anti-p21 human antibody (AF1047 R&D system). Then, the primary antibody was removed and washed 5 times for 5 min using a washing buffer of TBS with 0.1% Tween 20 (TBST). The blot was incubated with shaking for 4 h alongside the secondary antibody, an HRP conjugated goat anti-mouse IgG antibody, and an anti-rabbit IgG (HAF, R&D Systems) antibody at a 1:1000 dilution in TBS with 0.1% Tween 20 (TBST) and 5% BSA. The protein loading control, anti β-actin, was diluted to 1:5000 in TBS with 0.1% Tween 20 (TBST) and 5% BSA. The blot was rinsed 5 times for 5 min with TBST and incubated with shaking for 4 h alongside the anti-mouse IgG horseradish peroxidase conjugated secondary antibody (R&D Systems) at a dilution of 1:1000 in TBS with 0.1% Tween 20 (TBST) and 5% BSA. The blot was again rinsed with TBST, and the immunoreactive signal detections were visualized using a chemiluminescent HRP substrate (Clarity^TM^ Western ECL substrate, Bio-Rad, Hercules, CA, USA). The blot was then immediately exposed to the working solution for 5 min. All experiments were performed in duplicate.

## 5. Conclusions

Together, our results show that expanding the application of PARP inhibitors beyond breast cancer with *BRCA1* mutations (irrespective of *BRCA1* status) and optimizing combinative treatment using PARP inhibitors with antimetastasis ruthenium-based chemotherapy could represent a new therapeutic approach for TNBC harboring wild-type *BRCA1*. However, further animal studies and clinical trials are necessary to assess the efficacy and safety of the olaparib and RAPTA-T combination in treatments for this subtype of TNBC.

## Figures and Tables

**Table 1:** IC_50_ values (μM) of RAPTA-T and olaparib on MCF-7, MDA-MB-231 and MDA-MB-468 cells after treatment for 48 h. The values were expressed as mean ± SD of triplicate determination (*n* = 3).

Compound	IC_50_ (µM)		
	**MCF-7**	**MDA-MB-231**	**MDA-MB-468**
**RAPTA-T**	>1000	819.0 ± 6.9	484.9 ± 50.3
**olaparib**	82.7 ± 8.2	160.0 ± 26.5	86.5 ± 9.0
**RAPTA-T:olaparib** (Final concentration)	500:42	410:80	242:43
**Ratio** (RAPTA-T:olaparib)	12:1	5:1	6:1

## Data Availability

Data are available within the article. Reasonable inquiries for additional information can be directed to the corresponding author.
